# Molecular determinants of caste differentiation in the highly eusocial honeybee *Apis mellifera*

**DOI:** 10.1186/1471-213X-7-70

**Published:** 2007-06-18

**Authors:** Angel R Barchuk, Alexandre S Cristino, Robert Kucharski, Luciano F Costa, Zilá LP Simões, Ryszard Maleszka

**Affiliations:** 1 Faculdade de Filosofia, Ciências e Letras de Ribeirão Preto, Universidade de São Paulo, Ribeirão Preto, São Paulo, Brazil; 2 Instituto de Matemática e Estatística, Universidade de São Paulo, São Carlos, Brazil; 3 Instituto de Física de São Carlos, Universidade de São Paulo, São Carlos, Brazil; 4 Visual Sciences and ARC Special Research Centre for the Molecular Genetics of Development, Research School of Biological Sciences, Australian National University, ACT 0200, Canberra, Australia

## Abstract

**Background:**

In honeybees, differential feeding of female larvae promotes the occurrence of two different phenotypes, a queen and a worker, from identical genotypes, through incremental alterations, which affect general growth, and character state alterations that result in the presence or absence of specific structures. Although previous studies revealed a link between incremental alterations and differential expression of physiometabolic genes, the molecular changes accompanying character state alterations remain unknown.

**Results:**

By using cDNA microarray analyses of >6,000 *Apis mellifera *ESTs, we found 240 differentially expressed genes (DEGs) between developing queens and workers. Many genes recorded as up-regulated in prospective workers appear to be unique to *A. mellifera*, suggesting that the workers' developmental pathway involves the participation of novel genes. Workers up-regulate more developmental genes than queens, whereas queens up-regulate a greater proportion of physiometabolic genes, including genes coding for metabolic enzymes and genes whose products are known to regulate the rate of mass-transforming processes and the general growth of the organism (e.g., *tor*). Many DEGs are likely to be involved in processes favoring the development of caste-biased structures, like brain, legs and ovaries, as well as genes that code for cytoskeleton constituents. Treatment of developing worker larvae with juvenile hormone (JH) revealed 52 JH responsive genes, specifically during the critical period of caste development. Using Gibbs sampling and Expectation Maximization algorithms, we discovered eight overrepresented *cis*-elements from four gene groups. Graph theory and complex networks concepts were adopted to attain powerful graphical representations of the interrelation between *cis*-elements and genes and objectively quantify the degree of relationship between these entities.

**Conclusion:**

We suggest that clusters of functionally related DEGs are co-regulated during caste development in honeybees. This network of interactions is activated by nutrition-driven stimuli in early larval stages. Our data are consistent with the hypothesis that JH is a key component of the developmental determination of queen-like characters. Finally, we propose a conceptual model of caste differentiation in *A. mellifera *based on gene-regulatory networks.

## Background

Phenotypic variation among individuals of the same species triggered by environmental action is an intriguing biological phenomenon that can be found in quite striking manifestations in members of different insect orders [1]. In highly eusocial bees (Hymenoptera) one or a few females (queens) specialize in reproductive tasks, whereas a large number of quasi-sterile individuals (workers) engage in colony maintaining activities [2,3]. This polyphenism is generally determined by discrete switches during postembryonic development, and commences with the differential feeding of female larvae [4]. The nutritional stimuli trigger an endocrine response that is manifested by an elevated juvenile hormone (JH) titer in queen larvae when compared to workers [for review see [5]]. The queen-inducing properties of JH were first demonstrated by Wirtz and Beetsma [6], who topically applied JH on fourth and early fifth instar worker larvae [for similar results in stingless bees see [7,8]]. However, the molecular mechanisms underlying this phenomenon are not yet understood. In particular, we are largely ignorant of how nutritional factors affect the endocrine system and alter JH synthesis rates of queens and workers, and how these changes drive caste-specific developmental pathways during metamorphosis.

**Figure 1:**
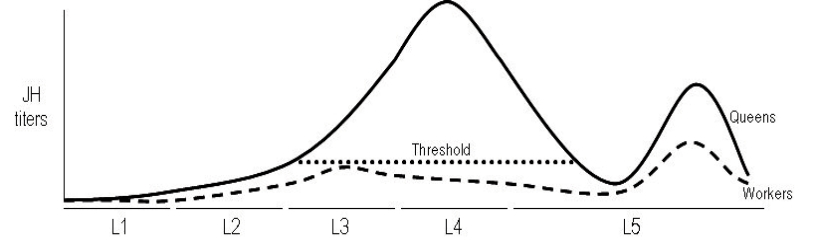
Reaching of the juvenile hormone (JH) threshold in developing females is proposed not only to allow for the general body growth and ovary development, but also to act by negatively regulating the development of some organismal systems that are characteristics of adult workers and are also present in the original developmental pattern. JH titres during larval development (L1–L5) data are modified from Hartfelder and Engels [5]

In *Apis mellifera*, a model system for caste development and division of labor in social Hymenoptera, young larvae of both castes are fed with royal jelly, a secretion produced by glands in the head of adult workers. Whereas these nurse bees feed copious amounts of royal jelly to queen larvae until they enter metamorphosis, they switch the diet for late instar worker larvae from pure royal jelly to a mixture of glandular secretions with honey and pollen (worker jelly). In addition, prospective queen larvae receive 10 times more food than worker larvae [9]. As a consequence of this differential feeding regime the two types of larvae follow two very different developmental trajectories, in spite of having exactly the same genetic background. Conceptually, this process of caste differentiation involves two kinds of alterations in the original developmental pattern (or ground plan present in ancestral solitary bees): one type, which we can call incremental alterations, affects the general growth of the body or specific organs, especially the ovaries. The other type can be considered as character state alterations that result in the presence or absence of entire specific structures, such as the pollen-collecting apparatus on the hind legs, wax glands, etc. Both types of alterations can be envisaged as JH threshold responses controlling the expression of genes involved in the development of specific organs and in specifying the general body plan (Figure 1).

The first large-scale study on the molecular biology of caste differentiation was done in *A. mellifera *by Severson et al. [10]. These authors demonstrated by *in vitro *translation analyses that queens and workers differ in their mRNA profiles during larval and prepupal stages. Later studies by Corona et al. [11] and Evans and Wheeler [12,13] found that most of the differentially expressed genes between prospective queens and workers were related to metabolic processes, and specifically, that queens up-regulate metabolic enzymes. Conversely, workers were shown to up-regulate a member of the cytochrome P450 family, hexamerin 2, dihydrodiol dehydrogenase and a fatty-acid binding protein. In addition, these studies revealed that several regulatory genes such as the mitochondrial translation initiation factor (AmIF-2mt), a member of the Ets family of transcription factors with a DNA binding domain, were also up-regulated in worker larvae [11].

In a recent study, Cristino et al. [14] examined the up-stream regulatory elements associated with all transcripts previously found to be differentially expressed in worker and queen larvae. They confirmed that the majority of the annotated differentially expressed genes (DEGs) are related to metabolic processes, with an interesting dichotomy for enzymes with hydrolase and oxidoreductase activities, which were found to be up-regulated in workers and queens, respectively. Genes up-regulated in workers were also shown to share more common (or conserved) overrepresented *cis*-elements when compared to genes up-regulated in queens.

While the aforementioned studies support the notion that incremental alterations are associated with the differential expression of physiometabolic genes, the nature of genetic mechanisms underlying the development of worker distinctiveness or character state alterations remains to be understood.

Here we used cDNA microarrays to monitor differential gene expression in honeybee queen and worker larvae and to identify *cis*-acting elements associated with these two developmental trajectories. Graph theory and complex networks concepts were adopted to attain an objective visual representation of the connectivity between motifs and genes in both castes. We identify groups of genes responsible for the development of queen and worker singularities and describe how their expression is co-regulated during critical stages of larval development. We also discuss the role of morphogenetic hormones in the developmental process of queen-like character and finally, propose a model of caste differentiation in *A. mellifera*.

## Results and Discussion

### Differential gene expression in developing queens and workers

We performed cDNA microarray analyses on a set of more than 6,000 unique genes throughout larval development of honeybees and identified a total of 240 genes as differentially expressed between queens and workers in the analyzed stages, namely L3, L4 and L5S2 (see Additional file 1; Methods and Gene Expression Omnibus database (NCBI), accession number GSE6452). Only genes that met stringent statistical criteria (α < 0.05; B > 0; see Methods) were selected as primary candidates.

Out of the 240 differentially expressed genes (DEGs), the majority (167) were up-regulated in fourth instar larvae (L4), 105 were up-regulated in prospective queens and 62 in worker-destined larvae [see Additional file 1]. Third instar larvae (L3) showed 37 DEGs and the fifth instar spinning stage larvae (L5S2) contributed with 36 DEGs [see Additional file 1]. This indicates that major changes in gene expression take place during the period of nutritional switch, in an environment with relatively high levels of JH for both queens and workers. Interestingly, out of 37 DEGs in L3 larvae the overwhelming majority (34) were found in workers, possibly reflecting the higher growth rate of young worker larvae [4]. Additional 52 genes were found to be up-regulated in JH-treated larvae. These ESTs most likely represent JH-responsive genes [see Additional file 1] because they reflect transcriptional changes occurring between 1 h and 24 hrs after hormone application.

Our functional classification of the honeybee DEGs based on *Drosophila melanogaster *Gene Ontology (GO) annotation (only reciprocal orthologs were considered) reveals that 56% of the queen up-regulated genes and 69% of the worker up-regulated genes do not have known counterparts in *Drosophila*. This predominance of novel (*Drosophila *unrelated) genes up-regulated in worker larvae has not been observed by Cristino et al. [14] who analyzed a small sample of 51 genes empirically selected as differentially expressed in queen and worker larvae [12,13,11,15].

Our finding that many of the up-regulated unique genes appear to be involved in the development of worker morphological and behavioral characteristics is compatible with the complexity of workers' body plan relatively to other insects such as *Drosophila*. When compared to honeybee queens, whose main purpose in life is to lay thousands of eggs, worker honeybees perform a myriad of activities inside and outside the colony and can even engage in reproductive tasks when released from the queen's inhibitory influence.

**Figure 2:**
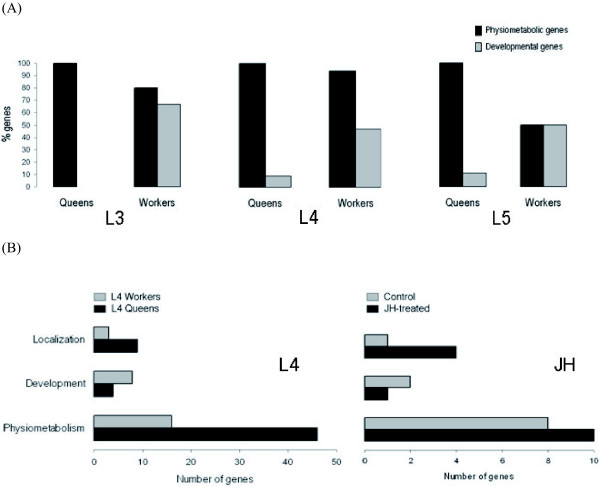
Functional trends of DEGs classified according to the Biological Process terms defined by GO consortium. (A) Developing workers up-regulate more developmental genes than queens in all studied larval instars. Physiometabolic genes are always more up-regulated than developmental genes (B) Juvenile hormone (JH) treatment induces a queen-like gene expression profile. Left panel: up-regulated genes in L4 queens/workers. Right panel: up-regulated genes in L4 Control/JH-treated workers. The proportion of Physiometabolic and Localization genes is higher in normal queens and JH-treated workers, whereas more Developmental genes are up-regulated in normal and in Control workers.

Based on GO terms for Biological Processes, we found that in all examined developmental stages workers up-regulate more developmental genes than queens (Figure 2A). The proportion of developmental genes is always around 50% in workers, whereas in queens this proportion is always very low, with a strong bias towards physiometabolic genes. For example, in L3 larvae nine developmental genes were found to be up-regulated in workers and none in queens and in L4 larvae there were eight such genes in workers and four in queens. Considering all developmental stages, workers up-regulated 17 genes classified as participating in developmental processes, whereas only five genes were up-regulated in queens. Interestingly, all these genes are classified as participating in developmental processes related to morphogenetic differentiation of specific organs, like pollen-collecting and reproductive apparati, nervous system, wax and Nasanov's glands, etc.

In agreement with previous work done in *Apis mellifera *[11-14] and in *Bombus terrestris *[16] we show that most of the known DEGs are related to physiometabolic processes (57 up-regulated in queen and 29 up-regulated in worker larvae). Among these, L4 queen larvae up-regulate five genes related to the metabolism of nitrogenous compounds (GB12123, GB13298, GB10789, GB10196, GB18599; see Additional file 1), while none of these genes appear to be up-regulated in workers. This is consistent with the fact that royal jelly is richer in nitrogen compounds (amino acids and nucleotides) than worker jelly [17,18]. On the other hand, in L3 stage there is only one nitrogen metabolism-associated DEG that is up-regulated in both castes, thus suggesting the existence of a maximization of feeding differences between developing queens and workers during L3–L4 stages. Furthermore, specifically during the L4 stage, queens up-regulate more genes associated with cellular localization, protein binding, nucleotide binding, nucleic acid binding, hydrolase and oxidoreductase activities than workers (Figure 2B). Proteins encoded by these genes are expected to participate in the physiometabolic processes leading to the differential growth of the queen's body.

Taken together, the relative proportion of differentially expressed physiometabolic genes is not unexpected, since most genes expressed during the honeybee life cycle are classified as belonging to three main categories; metabolic, cell growth and/or maintenance processes [see [19]]. This phenomenon is additionally aggravated in queen larvae, whose development is shifted towards a general growth.

### Differentially expressed genes related to the most conspicuous caste characteristics

Within the physiometabolic category there are some DEGs encoding metabolic enzymes and also genes whose products are known to regulate the rate of mass-transforming processes and the general growth of the organism (Table 1). Conversely, many genes that are well-characterized in *D. melanogaster *and in other model organisms may underlie processes leading to the development of caste-biased structures. For example, genes participating in neurogenesis, leg development, apoptosis, and genes coding for components of the cellular matrix (Table 1). The protein sequences of 81 DEGs clustered by functional groups were searched against a protein domains database (Pfam), as an additional support for the putative biological roles assigned by homology to the *Drosophila*'s counterparts (Table 1). The first three processes mentioned above are the basis for the respective morphological differences favoring the worker caste, thus defining the adult skills early in development: learning and memory, pollen and propolis collection, and a reduced reproductive capacity. The up-regulation in worker larvae of components of the cytoskeleton may reflect an early production of muscle elements, fundamental for the adult flight activities.

**Table 1:** DEGs clustered by their functional similarities based on GO annotation of *D. melanogaster *homologs and protein motifs described in Pfam database.

**Functional Group**	**Official_set_ID**	**Scaffold_ID**	**Gene_name**	**Flybase_ID**	**M**	**Pfam description**
**Growth (1)**						
**Protein folding (1.1)**						
	GB16563	Group6.34	Trap1-PA	CG3152	Q(1.00)	Hsp90 protein
						Histidine kinase-, DNA gyrase B-, and HSP90-like ATPase
	GB10587	Group13.6	Cct5-PA	CG8439	Q(0.95)	TCP-1/cpn60 chaperonin family
	GB12619	Group7.16	CG14894-PA	CG14894	Q(0.57)	Tetratricopeptide repeat
	GB12215	Group4.18	CG8863-PD	CG8863	Q(0.52)	DnaJ central domain (4 repeats)
**DNA binding (1.2)**						
	GB12948	GroupUn.293	His3.3A-PA	CG5825	Q(0.47)	Core histone H2A/H2B/H3/H4
	GB18508	Group2.39	skpA-PE	CG16983	Q(0.42)	Skp1 family, dimerisation domain
						Skp1 family, tetramerisation domain
	GB19338	Group10.29	crc-PB	CG8669	W(0.29)	bZIP transcription factor
	GB17426	Group3.15	Sirt6-PA	CG6284	W(0.48)	Sir2 family
**Endopeptidase (1.3)**						
	GB18440	Group13.4	Rpn6-PA	CG10149	Q(0.69)	PCI domain
	GB15218	Group5.21	Pros26.4-PA	CG5289	Q(0.40)	ATPase family associated with various cellular activities (AAA)
	GB11260	GroupUn.696	Rpn5-PA	CG1100	Q(0.38)	PCI domain
	GB19406	Group2.43	Rpn7-PA	CG5378	Q(0.43)	PCI domain
**Constituents of ribosome (1.4)**						
	GB15503	Group15.29	RpL4-PA	CG5502	Q(0.46)	Ribosomal protein L4/L1 family
	GB14287	Group7.1	mRpL45-PA	CG6949	Q(0.37)	Tim 44-like domain
	GB13769	Group8.29	mRpS22-PA	CG12261	Q(0.35)	--
**Protein binding(1.5)**						
	GB17469	Group4.23	shot-PG	CG18076	W(0.42)	Spectrin repeat
						Growth-Arrest-Specific Protein 2 Domain
						Plectin repeat
						EF hand
	GB11456	Group12.27	CREG-PA	CG5413	W(0.63)	--
	GB14059	GroupUn.5456	Nap1-PA	CG5330	Q(0.30)	Nucleosome assembly protein (NAP)
	GB19591	GroupUn.549	CG5742-PA	CG5742	Q(0.24)	Ankyrin repeat
**Other proteins (1.6)**						
	GB12179	Group3.19	Gyc76C-PC	CG8742	Q(0.18)	Adenylate and Guanylate cyclase catalytic domain
						Receptor family ligand binding region
						Protein kinase domain
						Protein tyrosine kinase
	GB13416	Group1.40	Trip1-PA	CG8882	Q(0.49)	WD domain, G-beta repeat
	GB16844	Group5.30	Ef1alpha100E-PC	CG1873	Q(0.62)	Elongation factor Tu GTP binding domain
						Elongation factor Tu C-terminal domain
						Elongation factor Tu domain 2
	GB18599	Group8.14	CG6287-PA	CG6287	Q(0.46)	D-isomer specific 2-hydroxyacid dehydrogenase, NAD binding domain
						D-isomer specific 2-hydroxyacid dehydrogenase, catalytic domain
	GB20148	Group8.12	pyd3-PA	CG3027	Q(1.60)	Carbon-nitrogen hydrolase
	GB13298	Group3.35	Gasp-PA	CG10287	Q(1.07)	Chitin binding Peritrophin-A domain
	GB18138	Group15.29	tws-PE	CG6235	Q(0.37)	WD domain, G-beta repeat
	GB11086	Group10.31	tor-PA	CG8274	Q(0.34)	TPR/MLP1/MLP2-like protein
	GB11665	GroupUn.5677	Idgf4-PB	CG1780	W(0.58)	Glycosyl hydrolases family 18

**Neurogenesis (2)**						
	GB17219	Group4.23	dac-PC	CG4952	W(0.28)	SKI/SNO/DAC family
	GB18802	Group2.40	Atx2-PB	CG5166	W(0.37)	Ataxin-2 N-terminal region
	GB17469	Group4.23	shot-PG	CG18076	W(0.42)	Spectrin repeat
						Growth-Arrest-Specific Protein 2 Domain
						Plectin repeat
						EF hand
	GB17380	Group3.22	fax-PC	CG4609	W(0.57)	
	GB14127	Group2.32	CRMP-PB	CG1411	W(1.25)	Amidohydrolase family
	GB12585	Group14.24	Eph-PE	CG1511	W(0.24)	Protein tyrosine kinase
						Ephrin receptor ligand binding domain
						Protein kinase domain
						Fibronectin type III domain
						SAM domain (Sterile alpha motif)
						GCC2 and GCC3
	GB19338	Group10.29	crc-PB	CG8669	W(0.29)	bZIP transcription factor
	GB14751	Group2.38	Tsp5D-PA	CG4690	W(0.44)	Tetraspanin family

**Leg development (3)**						
	GB18685	GroupUn.154	Gug-PC	CG6964	W(0.49)	Atrophin-1 family
						Myb-like DNA-binding domain
	GB17219	Group4.23	dac-PC	CG4952	W(0.28)	SKI/SNO/DAC family
	GB19338	Group10.29	crc-PB	CG8669	W(0.29)	bZIP transcription factor
	GB18802	Group2.40	Atx2-PB	CG5166	W(0.37)	Ataxin-2 N-terminal region

**Apoptosis and ovary dev. (4)**						
**Protein binding (4.1)**						
	GB16563	Group6.34	Trap1-PA	CG 3152	Q(1.0 0)	Hsp90 protein
	GB10850	GroupUn.303	l(2)tid-PC	CG5504	Q(0.42)	DnaJ C terminal region
						DnaJ domain
						DnaJ central domain (4 repeats)
	GB18249	Group4.15	eff-PA	CG7425	Q(0.36)	Ubiquitin-conjugating enzyme
	GB10667	Group15.32	Gl-PA	CG9206	Q(0.41)	CAP-Gly domain
**Other proteins (4.2)**						
	GB16903	Group8.23	cathD-PA	CG1548	W(0.99)	Eukaryotic aspartyl protease
	GB18912	Group1.14	26-29-p-PA	CG8947	W(0.49)	Papain family cysteine protease
						Cathepsin propeptide inhibitor domain (I29)
	GB15106	Group13.9	CG11159-PA	CG11159	W(0.57)	C-type lysozyme/alpha-lactalbum in family
	GB18802	Group2.40	Atx2-PB	CG5166	W(0.37)	Ataxin-2 N-terminal region
	GB10394	Group3.12	Traf1-PA	CG3048	W(0.36)	TRAF-type zinc finger
						MATH domain
	GB11086	Group10.31	tor-PA	CG8274	Q(0.34)	TPR/M LP1/M LP2-like protein

**Cytoskeleton and others (5)**						
**Cytoskeleton proteins (5.1)**						
	GB11965	Group8.46	Mhc-PB	CG17927	W(0.36)	Myosin tail
						Myosin head (motor domain)
	GB17681	Group7.42	Act5C-PB	CG4027	W(0.38)	Actin
	GB16881	Group6.3	up-PG	CG7107	W(0.30)	--
	GB17469	Group4.23	shot-PG	CG18076	W(0.42)	Spectrin repeat
						Growth-Arrest-Specific Protein 2 Domain
						Plectin repeat
						EF hand
	GB19016	Group1.21	lva-PC	CG6450	W(0.52)	--
	GB10337	Group4.18	Ank2-PB	CG7462	W(0.61)	Ankyrin repeat
						ZU5 domain
						Death domain
**Transcription associated prot. (5.2)**						
	GB15645	Group11.31	NFAT-PA	CG11172	W(0.71)	Rel homology domain (RHD)
						IPT/TIG domain
	GB16648	Group9.18	usp-PA	CG4380	W(0.20)	Ligand-binding domain of nuclear hormone receptor
						Zinc finger, C4 type (two domains)
	GB19023	Group2.33	Hcf-PD	CG1710	W(0.42)	Kelch motif
						Fibronectin type III domain
**Other proteins (5.3)**						
	GB10771	GroupUn.3863	Jheh1-PA	CG15101	W(0.46)	Epoxide hydrolase N terminus
						alpha/beta hydrolase fold
	GB11059	Group8.20	RfaBp-PA	CG11064	W(0.49)	Lipoprotein amino terminal region
						von Willebrand factor type D domain
						Domain of Unknown Function (DUF1081)
	GB17439	Group3.31	Scs-fp-PC	CG17246	W(0.59)	FAD binding domain
						Fumarate reductase/succinate dehydrogenase flavoprotein C-terminal domain
	GB16882	Group16.12	wal-PA	CG8996	Q(0.51)	Electron transfer flavoprotein FAD-binding
						Electron transfer flavoprotein domain
	GB12827	GroupUn.5348	Dox-A2-PA	CG10484	Q(0.42)	PCI domain
	GB10658	Group14.24	l(3)neo18-PA	CG9762	W(0.70)	--

**JH responsive (6)**						
**Metabolic enzymes (6.1)**						
	GB19085	Group13.10	CG9009-PA	CG9009	Q(1.10)	AM P-binding enzyme
	GB16747	Group4.23	CG17323-PA	CG17323	Q(0.46)	UDP-glucoronosyl and UDP-glucosyl transferase
	GB15619	Group2.43	CG8036-PC	CG8036	Q(0.33)	Transketolase, thiamine diphosphate binding domain
						Transketolase, pyridine binding domain
						Transketolase, C-terminal domain
	GB13982	Group13.10	Faa-PA	CG14993	W(0.34)	Fumarylacetoacetate (FAA) hydrolase family
	GB14870	Group13.13	CG31472-PB	CG31472	W(0.38)	Pyridoxamine 5'-phosphate oxidase
	GB13626	GroupUn.593	CG6206-PB	CG6206	W(0.40)	Glycosyl hydrolases family 38 N-terminal domain
						Glycosyl hydrolases family 38 C-terminal domain
	GB19030	Group15.25	CG10638-PA	CG10638	W(0.60)	Aldo/keto reductase family
	GB19460	Group1.18	Ald-PB	CG6058	W(0.86)	Fructose-bisphosphate aldolase class-I
	GB17864	GroupUn.36	CG7920-PA	CG7920	W(0.90)	Acetyl-CoA hydrolase/transferase
	GB13401	Group6.55	CG9629-PA	CG9629	W(1.70)	Aldehyde dehydrogenase family
**Carrier activity (6.2)**						
	GB14751	Group2.38	Tsp5D-PA	CG4690	W(0.44)	Tetraspanin family
	GB12135	Group1.65	CG1213-PC	CG1213	Q(0.76)	Sugar (and other) transporter
						Major Facilitator Superfamily
	GB13781	Group15.34	CG33528-PC	CG33528	Q(0.58)	Major Facilitator Superfamily
	GB17499	Group7.37	sesB-PC	CG16944	Q(0.57)	Mitochondrial carrier protein
**Protein binding (6.3)**						
	GB13815	Group11.41	Cdep-PC	CG31536	Q(0.72)	FERM domain (Band 4.1 family)
						PH domain
	GB18087	Group1.59	Nacalpha-PC	CG8759	Q(0.47)	NAC domain
	GB10836	Group2.22	Hsc70Cb-PC	CG6603	Q(0.44)	Hsp70 protein
	GB19860	Group1.82	Hsc70-5-PA	CG8542	Q(0.42)	Hsp70 protein
	GB18197	Group16.15	CAP-PC	CG18408	Q(0.53)	SH3 domain
	GB10587	Group13.6	Cct5-PA	CG8439	Q(0.31)	TCP-1/cpn60 chaperonin family
**Nucleic acid binding (6.4)**						
	GB15755	Group5.12	RpS11-PC	CG8857	Q(0.61)	Ribosomal protein S17
	GB15503	Group15.29	RpL4-PA	CG5502	Q(0.39)	Ribosomal protein L4/L1 family
	GB20034	GroupUn.1172	CG8108-PB	CG8108	Q(0.32)	--
**Other proteins (6.5)**						
	GB12348	Group12.16	CG31952-PA	CG31952	Q(0.91)	--
	GB14145	Group3.14	uzip-PA	CG3533	Q(0.49)	--
	GB19075	Group5.26	CG3153-PA	CG3153	Q(0.28)	ML domain
	GB19923	Group16.6	Cp1-PC	CG6692	W(0.42)	Papain family cysteine protease
						Cathepsin propeptide inhibitor domain (I29)
	GB11059	Group8.20	RfaBp-PA	CG11064	W(0.74)	Lipoprotein amino terminal region
						von Willebrand factor type D domain
						Domain of Unknown Function (DUF1081)
	GB18626	GroupUn.41	Ccp84Ad-PA	CG2341	Q(1.53)	Insect cuticle protein

**Hormone + caste (7)**						
JH24htreat+QL4	GB19085	Group13.10	CG9009-PA	CG9009	Q(1.1)	AMP-binding enzyme
JH24htreat+QL4	GB10836	Group2.22	Hsc70Cb-PC	CG6603	Q(0.44)	Hsp70 protein
JH24htreat+QL4	GB19860	Group1.82	Hsc70-5-PA	CG8542	Q(0.42)	Hsp70 protein
JH24htreat+QL4	GB15503	Group15.29	RpL4-PA	CG5502	Q(0.39)	Ribosomal protein L4/L1 family
JH24htreat+QL4	GB15619	Group2.43	CG8036-PC	CG8036	Q(0.33)	Transketolase, thiamine diphosphate binding domain
						Transketolase, pyridine binding domain
						Transketolase, C-terminal domain
JH24htreat+QL4	GB10587	Group13.6	Cct5-PA	CG8439	Q(0.31)	TCP-1/cpn60 chaperonin family
JH24hcontrol+WL4	GB13401	Group6.55	CG9629-PA	CG9629	W(1.70)	Aldehyde dehydrogenase family
JH24hcontrol+WL4+WS2	GB16686	Group15.25	no similarity		W(0.96)	--
JH24hcontrol+WL4	GB16631	Group16.12	no reciprocal		W(0.74)	Leucine Rich Repeat
JH24hcontrol+WL4	GB11059	Group8.20	RfaBp-PA	CG11064	W(0.44)	Lipoprotein amino terminal region
						von Willebrand factor type D domain
						Domain of Unknown Function (DUF1081)

M = log2 of the gene expression differences between developing queen and worker samples (based on microarray experiments). W = worker, Q = queen

With regard to hormonal control of larval development, the experiment with JH treatment of developing worker larvae allowed us to identify 52 'JH-responsive' genes involved either directly or indirectly in JH signaling, specifically during the critical period of caste development. Ten DEGs that were identified in both JH and caste assays were clustered as "JH responsive + caste" genes (Table 1). Six genes were found in both JH treated larvae and L4 queens, and four genes were held in common between JH controls and L4 workers.

#### Genes for differential growth

Queens up-regulate more physiometabolic genes than workers. Moreover, a vast majority of these genes code for products with protein-binding activities like chaperonins or chaperon binding proteins and ribosomal and proteasome related proteins with endopeptidase activities (Table 1).

A fundamental key controller of physiometabolic processes, also up-regulated in L4 queens, is a member of the insulin-signaling pathway *tor*. This conserved gene regulates translation initiation in response to nutrients from yeast to mammals. In the insect fat body, Tor acts as a sensor of amino acid levels in the hemolymph [20]. When there are sufficient amino acids available, the activation of the Tor system triggers the expression of ALS protein (mammalian acid labile subunit ortholog), a member of a systemic communicational pathway, which signals to other larval tissues, in a PI3K-dependent manner, the nutritionally favorable situation of the organism [21]. In *corpora allata*, the activation of Tor pathway could trigger the synthesis of juvenile hormone, one of the central regulators of insect development. Other responsive organs are the ovaries, where cell proliferation has been demonstrated to be responsive to the insect's nutritional environment [22].

Acting together with Tor in determining differential growth between queens and workers are CREG and CRC (Table 1). CREG (cellular repressor of E1A-stimulated genes), that is up-regulated in L4 workers, is a secreted glycoprotein. In humans, it antagonizes cellular transformation by E1A and ras [23]. CRC encodes a *Drosophila *homolog of vertebrate ATF4, a member of the CREB/ATF family of basic-leucine zipper (bZIP) transcription factors. This protein (CRC) participates in a conserved mechanism of sensing amino acid deprivation and stress induction [24]. Thus, the up-regulation of *tor *in prospective queen larvae and of *creg *and *crc *in prospective worker larvae may constitute a dual system of growth determination in response to differential feeding in honeybees.

#### Genes for differential neurogenesis

Learning and memory-related skills that honeybee workers use for navigation, foraging, nestmate recognition, and other activities are believed to be associated with the prominent regions of insect brains, the mushroom bodies [25]. As expected, the ratios typically used to evaluate the relative size of specific brain areas versus the body size support the notion that workers have bigger and more developed mushroom bodies than queens [26,2]. We found that five genes encoding proteins that participate in neural system development in *Drosophila *and in vertebrates (*dac, atx2, shot*, *ephR *and *fax*) are up-regulated in developing worker larvae (Table 1). Since the nervous system in queens and workers begins to differentiate during post-embryonic stages [26] these five genes are candidates for molecular determinants of the observed morphological differences in developing brains.

#### Genes for differential leg development

The making of an insect leg is a complex process that is even more complicated in worker honeybees because of the presence of unique structures for pollen and propolis collection [27]. We found three genes to be up-regulated in L3 workers that have been shown to act as regulators of leg development (*gug, dac *and *crc*) and one gene regulating bristle morphology (*atx2*; Table 1) in *Drosophila *[28]. This finding is consistent with the importance of bristles morphology and density for the pollen-collecting apparatus [27]. The temporal expression of these genes during leg imaginal disc development in the critical period of caste differentiation suggests that they have been recruited, together with other (unknown) genes, to regulate the alternative leg structures in *A. mellifera *castes.

#### Genes for differential ovary development

Programmed cell death (PCD) is a process that, in concert with cell proliferation, modulates the development of specific organs during metazoan ontogenesis. In insects, this process is commonplace during larval nervous system development and during metamorphosis, where major systems reorganization occurs. In the honeybee, PCD in the worker ovary reduces the number of ovarioles during metamorphosis from 150–200 primordia to less than 10. It has been shown that this phenomenon is prevented from occurring in queen larvae by high titres of JH [29] that may inhibit the ecdysteroid-triggered PCD, as suggested for *Drosophila *[30]. As shown in Table 1, several genes preferentially expressed in worker larvae are associated with PCD. Among these are the cathepsin D gene (*cathD*) [31], and a gene coding for a lysozyme that participates in autophagic cell death of salivary glands in *Drosophila*, CG11159 [32]. We also found *atx2 *(Ataxin-2) gene up-regulated in L3 workers. The transgenic over-expression of *atx2 *in *Drosophila *results in female sterility, possibly by impairing the adhesion between oocytes and follicular cells [33]. Another example of genes up-regulated in worker larvae is *traf1 *(TNF-receptor-associated factor 1). Its product participates in both autophagic cell death and induction of apoptosis in *Drosophila*. Several genes up-regulated in L4 queens are similar to *Drosophila *and human genes coding for proteins with anti-apoptotic activity. One of them is *lethal*(2) *tumorous imaginal discs *(*l(2)tid; *34). Another one is *Trap1*, an unfolded protein binding protein and a member of the heat-shock family of proteins that may play an important role in the suppression of apoptosis caused by formation of ROS [35]. In addition, *tor*, which is also up-regulated in L4 queens, is a negative regulator of autophagic cell death [36]. Thus, when activated by factors acting downstream of the insulin receptor, Tor could provide a link between nutrient availability and the regulation of tissue disintegration, such as in the ovarian tissue of honeybees. Taken together, all these genes are likely to participate in the regulatory processes underlying the differential reproductive capacity of honeybee queens and workers.

#### Other genes differentially expressed between developing queens and workers

Several DEGs that are up-regulated in worker larvae code for cytoskeleton constituents (Table 1). Among these are genes encoding myosin II heavy chain (*mhc*), actin, (*act5C*), troponin T, and the genes *upheld, shot, lva *and *ank2*. In honeybees, muscle development continues until the early fifth instar allowing for the replacement of larval muscles by imaginal muscles during metamorphosis [27]. One explanation for this increased expression of several cytoskeletal genes associated with muscle development may be the differential flight ability of adult honeybee workers. The differential expression of muscle related genes has also been observed in a closely related stingless bee, *Melipona quadrifasciata *[37]. Finally, L3 workers differentially express the *usp *(*ultraspiracle*) gene that codes for the ecdysone receptor partner and a strong candidate for a JH receptor in insects [38], including honeybees [39].

### Juvenile hormone induces queen-like characteristics by preferentially up-regulating the expression of physiometabolic genes

Inspired by the widely known effect of JH on caste differentiation in honeybees [6] we examined gene expression in worker larvae following the application of exogenous JH-III. We found four genes (*ccp84Ad *and three unknown ones) to be up-regulated in L4 larvae 1 h after JH treatment against three up-regulated genes (*tsp5D *and two unknown ones) in control L4 workers. *Ccp84Ad *that is highly expressed in L4 queens is annotated as a structural component of larval cuticle [40]. Since L4 queen larvae have high titres of endogenous JH [5], this rapid and positive regulation of *ccp84Ad *by JH suggests that this is an early responsive gene in the expression cascade promoted by JH. *Tsp5D*, a tetraspanin protein gene, is up-regulated in control L4 workers. A member of the tetraspanin family of proteins resides in cell surfaces of growth cones facilitating synapse formation during *Drosophila *neurogenesis [41]. Thus, since queen larvae have higher titres of JH, the repression by exogenous JH of the expression of a gene (*tsp5D*) that participates in cone growing, makes this protein a candidate player in worker neurogenesis.

**Figure 3:**
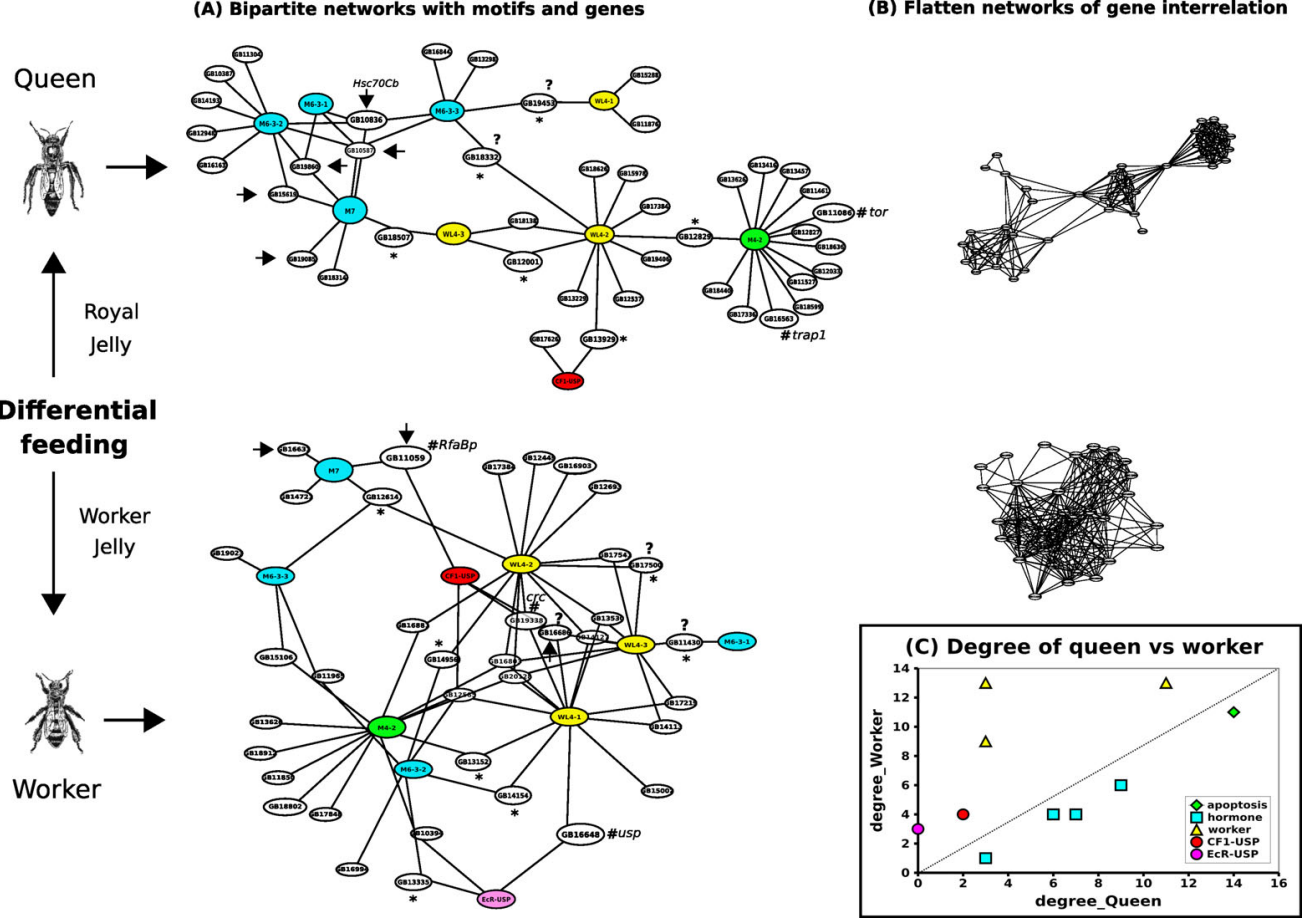
Networks depicting putative gene interactions based on the occurrence of overrepresented motifs in the UCR of DEG between *A. mellifera *castes. (A) Bipartite graph representing the occurrence of motifs (colorized circles) in the UCR of DEG in queen and worker castes. Motifs represented in blue were found in the functional group "JH responsive" (M6-3-1, M6-3-2, M6-3-3) and "hormone+caste" (M7), those in green were found in the functional group "apoptosis/other proteins" (M4-2), in yellow in top10-WL4 genes (WL4-1, WL4-2, WL4-3) and in magenta are motifs found experimentally in other insects (CF1-USP and EcR-USP). The black arrows point to genes coherently up-regulated in caste stages and JH assay. Genes with unknown function are marked by a question mark (?). Genes marked by an asterisk (*) were not in the training dataset for motif discovery. The worker DEG marked by a hash (#) are *usp, crc *and *RfaBp*, repressed by hormones. The queen DEGs marked by a hash (#) are *tor *and *trap1*, negative regulators of cell death in response to nutritional availability. (B) One layer graph (subsumed) designed to obtain measures of complex networks. Clustering coefficient (cc) and degree (d) show that worker's network (d = 62.21 ± 28; cc = 0.37 ± 0.23) is more interconnected than queen's network (d = 31.23 ± 15.67; cc = 0.36 ± 0.25). This suggests the worker DEGs share much more conserved *cis*-elements when compared to queen DEGs. (C) A plot obtained by representing each motif by a point with abscissa equal to its degree in the queen network and the ordinate equal to its degree in the case of the worker network. The fact that most nodes resulted above the main diagonal line (represented by the dashed line) objectively indicates that most promoters, except for "hormone" and "apoptosis" motifs, regulate more genes in the latter case (workers).

Among the 45 DEGs identified as up-regulated at 24 h after JH treatment, 28 were found to be induced and 17 repressed by this hormone. Interestingly, the majority of JH-responsive genes with known orthologs in *Drosophila *are physiometabolic genes (Figure 3). The exceptions are *unzipped *(*zip*), which participates in axonogenesis processes [42], *cathepsin L *(*Cp1*), implicated in cell death processes [32] and embryonic *aldolase *[43]. Out of the 45 JH responsive genes, 26 have known *Drosophila *orthologs.

Previous studies have shown that JH treatment accelerates behavioral maturation in young bees [44] and plays a role as an organizer of the mushroom bodies [45]. Recently, a microarray analysis was used to understand the effects of a JH analog (Methoprene) on brain gene expression profiles during behavioral maturation of honeybee workers [46]. Methoprene induces significant forager-like changes in gene expression even in workers with no foraging experience suggesting that the increase in JH titres may be related to expressional changes occurring during the natural transition from hive to forager behavior [47,46]. Interestingly, 17 out of 52 JH responsive DEGs identified in our work (JH 1 h and JH 24 h) in worker L4 larvae overlap with DEGs in the brain of adult workers treated with Methoprene in the study reported by Whitfield et al. [46]. The effects of JH-like compounds caused the same shift in gene expression for 10 DEGs in both assays. Of particular interest are two DEGs considered as markers for behavior, namely *Hsc70Cb *(GB10836; a forager marker) and *RfaBp *(GB11059; a nurse marker) [48,46]. In our study, *Hsc70Cb *and *RfaBp *were up-regulated in queen larvae and in worker larvae, respectively. Furthermore, these two DEGs change their expression profiles in a hormone-dependent manner as observed in worker L4 larvae (our work) and young adult worker bees [46]. *RfaBp *transcription was down-regulated by JH treatment in two very different stages of bee development (caste determination in larvae and 'hive-bee-to-forager transition' in adults). On the other hand, *Hsc70Cb *was up-regulated by JH treatment in worker L4 larvae as observed in queen L4 larvae (high titres of JH) but was down-regulated by Methoprene treatment in young adult bees [46]. The contrasting effects of JH on *Hsc70Cb *expression during different life stages are interesting examples of how hormones can exert their regulatory effects on genes expression in a context-dependent manner. Until recently, only a small number of insect genes had been described as being either directly or indirectly regulated by JH [39,49]. Consequently, this study and the reported by Whitfield [46] represent a significant expansion of this functional category.

Finally, and as shown in Figure 2(B), JH treatment induces a queen-like expressional pattern. Physiometabolic and Localization genes are up-regulated in JH-treated workers, whereas Developmental genes are up-regulated in control workers. These results support the notion that JH is a potent activator of physiometabolic processes and exerts its role by inducing incremental alterations.

### Motif analysis in the upstream control regions suggests that some genes are co-regulated

Most of the genes identified as differentially expressed between the two castes have been assigned to functional groups according to the literature and functional databases (GO and Pfam). We hypothesized that the groups of DEGs sharing common expression patterns might be used to infer putative clusters of co-regulated genes by means of computational analyses of DNA-sequence motifs [50-52].

A motif discovery pipeline was run on 19 sets of functional groups (Table 1) and on two sets of top10 DEGs observed in the L4 stage (Table 2). Nine sets of genes show significant differences in the distribution of motif scores when compared to control (random) sets [see Additional file 2]. The datasets neurogenesis (2), leg development (3), apoptosis/other proteins (4.2), cytoskeleton/cytoskeleton proteins (5.1), cytoskeleton/transcription associated proteins (5.2), cytoskeleton/other proteins (5.3), JH responsive/protein binding (6.3), hormone+caste (7) and top10-WL4 (9) show evidences for the occurrence of more conserved motifs than expected by chance, but only 8 motifs were considered as overrepresented (Church <= 1e-10 and ROC-AUC >= 0.7) in 4 functional groups [Table 3; 1 motif in (4.2), 3 motifs in (6.3), 1 motif in (7) and 3 motifs in (9)]. No similarity was found by aligning these eight motifs with *D. melanogaster *binding site sequences described in the TRANSFAC database [53].

**Table 2:** Top10 most DEGs in L4 developmental stage of queens and workers ranked by their M values.

**Caste DEG L4**	**Official_set_ID**	**Scaffold_ID**	**Gene_name**	**Flybase_ID**	**M**
**Queen (QL4)**	GB18626	GroupUn.41	Ccp84Ad-PA	CG2341	1,44
	GB11403	Group12.30	CG6414-PA	CG6414	1,18
	GB13298	Group3.35	Gasp-PA	CG10287	1,07
	GB18599	Group8.14	CG6287-PA	CG6287	1,00
	GB16563	Group6.34	Trap1-PA	CG3152	1,00
	GB17384	GroupUn.41	n.d.	n.d.	0,96
	GB19671	Group11.8	CG9200-PA	CG9200	0,95
	GB10587	Group13.6	Cct5-PA	CG8439	0,95
	GB10836	Group2.22	Hsc70Cb-PC	CG6603	0,92
	GB16452	GroupUn.1387	CG5525-PA	CG5525	0,88

**Worker (WL4)**	GB19820	Group14.17	n.d.	n.d.	-1,78
	GB14127	Group2.32	CRMP-PB	CG1411	-1,25
	GB14113	GroupUn.8421	Mi-2-PA	CG8103	-1,18
	GB16803	Group14.17	n.d.	n.d.	-1,01
	GB15002	Group9.25	CG31711-PA	CG31711	-0,99
	GB16903	Group8.23	cathD-PA	CG1548	-0,99
	GB20128	Group16.4	CG4040-PA	CG4040	-0,85
	GB13530	Group2.32	CG30105-PA	CG30105	-0,83
	GB16686	Group15.25	n.d.	n.d.	-0,78
	GB17541	Group15.29	CG5059-PA	CG5059	-0,77

M = log2 of the gene expression differences between developing queen and worker samples (based on microarray experiments). (-) up-regulated in workers. n.d.: not determined.

**Table 3:** Overrepresented motifs found in three functional groups and in the top10 DEGs of worker L4.

**Functional groups**	**Motif_name**	**Motif_consensus**	**MAP**	**Church**	**ROC_AUC**	**MNCP**
**Apoptosis and ovary dev.(4)**						
Other proteins (4.2)**						
	M4-2	TwTG.GAAAAkwr.AkA	7.89	5.32e-10	0.90	147.42

**JH responsive (6)**						
Protein binding (6.3)**						
	M6-3-1	yCwcgcwt..Gy..Gww.GA	11.22	8.48e-13	0.91	180.52
	M6-3-2	kSGtGmaw.....GMr..am	12.20	5.32e-10	0.91	216.45
	M6-3-3	C.wCG.aCGA..m.Kw.kA	7.80	5.59e-10	0.74	243.93

Hormone + caste(7)**						
	M7	KAtwCr..k.g.w....CG.Gm	11.46	1.72e-10	0.79	92.14

Top10 DEG L4 Worker*						
	WL4-1	gwms.aGmsG......CGs.g	45.87	9.32e-13	0.74	36.53
	WL4-2	cwsAsw.GCGYry	9.39	1.72e-10	0.78	74.80
	WL4-3	G.SG....m.CRr.g.....s..wg	15.11	1.72e-10	0.74	97.93

* P < 0.05; ** P < 0.01 for the four metrics.

The upstream control regions (UCRs) of worker top10 genes have significantly more overrepresented DNA-sequence motifs than queen top10 genes (Table 3; three motifs in workers and none in queens). This result is in agreement with Cristino et al. [14] who reported that the number of overrepresented motifs discovered in worker genes was much higher than in queen genes. Interestingly, at least two of the discovered motifs were found in UCRs of 13 genes (six in queens and seven in workers, Figure 3) that were not part of the dataset used in the motif discovery pipeline. Two queen DEGs showed putative binding sites for CF1-USP (GB12001, GB13929; 54). In workers, a group of four DEGs has binding sites for CF1-USP and three DEGs for EcR-USP (55; Figure 3).

The main interactions among the queen DEGs are around the motifs discovered in JH responsive datasets (Table 1; Figure 3). Interestingly, *Hsc70Cb *gene has all four hormone-related motifs (M6-3-1, -2, -3 and M7; Figure 3) in its UCR. On the other hand, worker DEGs are largely organized around the motifs found in the top10-WL4 dataset (WL4-1, -2, -3; Table 3, Figure 3). The localization of three up-regulated genes, GB16648 (*usp*), GB19338 (*crc*) and GB11059 (*RfaBp, Retinoic and Fatty acid Binding Protein*), in the worker's network (Figure 3) is particularly informative. The motifs for the binding of EcR-USP and the best motif of the top10-WL4 dataset (WL4-1; Table 3) were found in the UCR of *usp *itself, indicating that this gene can be regulated by a protein that is important for transcriptional control in workers and also by its own protein when heterodimerized with EcR, as suggested by Barchuk et al. [39]. In *crc *gene, up-regulated in workers, we found three motifs, CF1-USP and two top10-WL4 motifs (WL4-1 and WL4-2; Table 3 and Figure 3), suggesting that *crc *can be regulated by USP and other regulatory proteins important for worker pattern development. *RfaBp *gene was also found to be up-regulated in workers by Evans and Wheeler [12,13] and we show that it is up-regulated in worker larvae and responds negatively (it is down-regulated) to JH treatment. In adult workers, *RfaBp *expression is repressed by a JH analog [46] and has been considered a nurse marker gene [48]. Two motifs were found in the *RfaBp *UCR, CF1-USP and the best motif of the "Hormone+caste" dataset (M7, Table 3 and Figure 3), suggesting that *RfaBp *can be regulated by USP and also by another regulatory protein involved in JH response. Taken together, the available data indicate that these caste determining genes (*RfaBp*, *crc *and *usp*) seem to play an additional important role in regulating major phenotypic changes in honeybee development.

After searching for matches of at least 60% similarity to the discovered motifs and the two canonical patterns, CF1-USP and EcR-USP, for the UCRs of 183 DEGs (105 for queens, 78 for workers), we designed putative transcriptional networks based on the occurrence of overrepresented motifs in the UCR of DEGs between *A. mellifera *castes (Figure 3). The first type of network considered here, illustrated in Figure 3(A) is a bipartite network involving both motifs and genes. The second type of network, illustrated in Figure 3(B) depicts only genes and their interrelationship. Figure 3(C) shows that most motifs, except for "hormone" and "apoptosis" motifs, are plotted above the main diagonal line (dashed line) and regulate more genes in workers.

The similar clustering coefficients obtained for workers (cc = 0.37 ± 0.23) and queens (cc = 0.36 ± 0.25) indicates similar dense connectivity among the immediate neighbors of each gene. Nevertheless, the degree measure shows that the worker network is substantially denser (d = 62.21 ± 28) showing more interconnections than that obtained for queens (d = 31.23 ± 15.67), which has also a more modularized system (Figure 3B). In agreement with Cristino et al. [14], this result indicates that workers genes are more strongly interrelated (Figure 3). Moreover, the obtained networks show that worker DEGs share significantly more conserved motifs than expected by chance.

All the results presented here suggest the existence of groups of co-regulated genes and highlight potential key genes (*tor, usp*, *crc*, *RfaBp*) which might determine developmental processes leading to the formation of caste-specific morphogenetic fields.

## Conclusion

### Towards a unified model of caste differentiation in the honeybee

**Figure 4:**
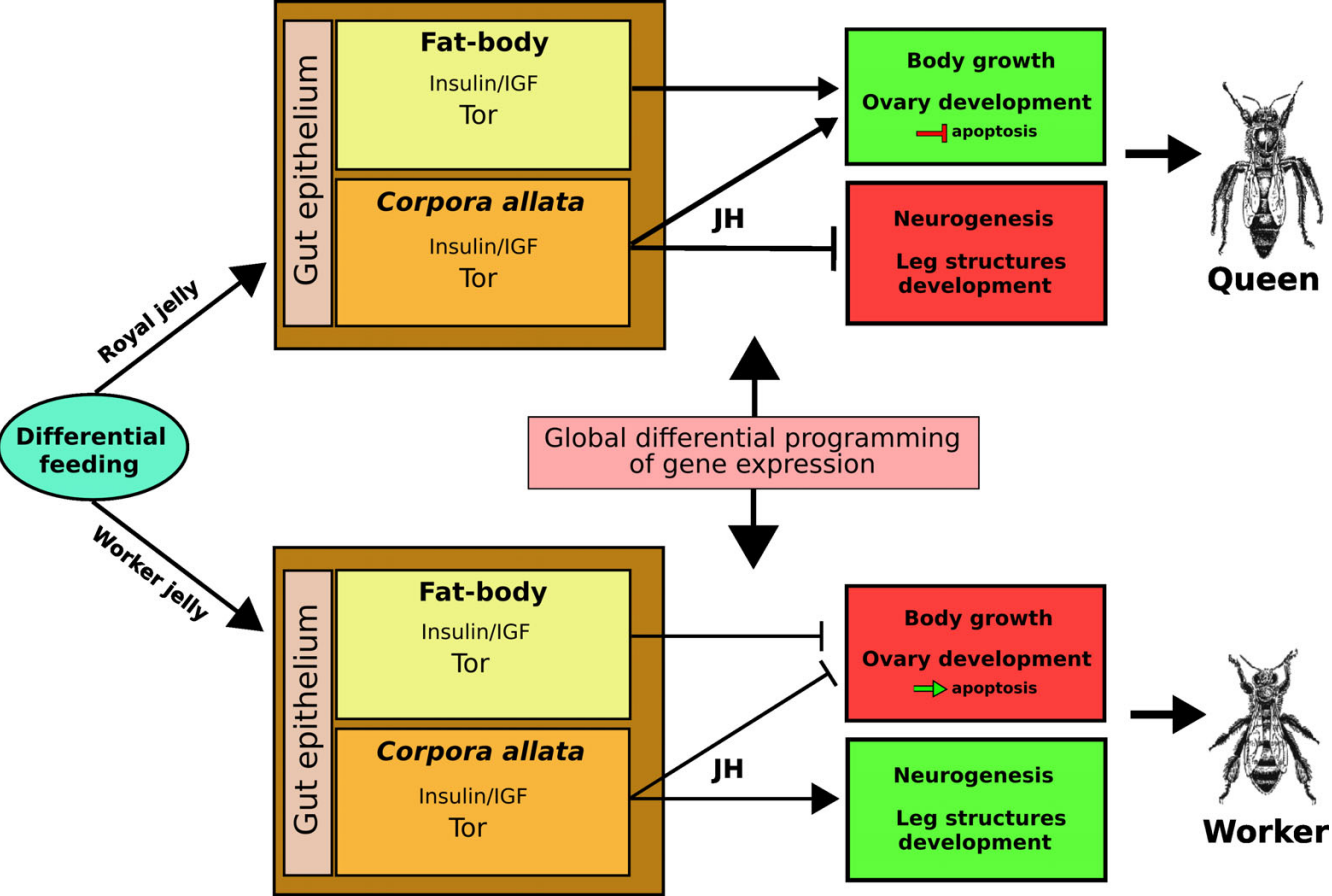
Proposed general model of caste differentiation in *Apis mellifera*. Arrows thickness indicates the relative action levels of the considered factors. Recent studies by our group suggest that the global differential programming of gene expression in the honeybee is controlled by DNA methylation mechanism in a manner similar to epigenetic transcriptional changes inducible by environmental factors in vertebrates (Maleszka et al., in preparation). For details see Section "Towards a unified model of caste differentiation in the honeybee".

In an attempt to consolidate our results with published data, we propose the following model for caste differentiation in *A. mellifera *(Figure 4). The type of food eaten by the larva must first be recognised by the receptor system in the gut epithelial cells followed by complex signalling via the stomatogastric nervous system [[56]; Maleszka et al., in preparation] that sends the information to the brain and the retrocerebral endocrine system. *Corpora allata *(CA) activity and the behavior of target tissues may be even under the upstream regulation of insulin/IGF molecules eventually secreted by the neurosecretory cells of the larval brain [57], as suggested for *Drosophila *[see [58]]. The other sensing organ, the fat body (FB), receives the information directly from the hemolymph. Thus, high level inputs in these organs in prospective queens result in the activation of the insulin/IGF pathway and the Tor system, which in turn increases the levels of JH synthesis in the CA, and may trigger the ALS-mediated systemic communicational pathway in FB. "Worker jelly", on the other hand, affects insulin/IGF pathway in a less pronounced manner, and may not be able to increase the levels of JH above a specific threshold.

As a result of the activated signaling pathways, the high titres of JH in prospective queens regulate the expression of physiometabolic genes that together with the available nutrients from royal jelly determine the general body growth pattern. In this model, the up-regulation of *tor *in prospective queen larvae can be seen as a determinant of the observed differential growth rates. On the other hand, low levels of JH combined with limited nutrient availability in prospective workers lead to the development of smaller adults. In this case, the up-regulation of *creg *and *crc *(negative growth regulators) in workers may constitute the second determinant of a dual system of growth regulation in response to differential feeding in honeybees.

Furthermore, reaching a JH threshold in prospective queens (Figure 1) not only permits general body growth but also acts by negatively regulating the development of some organismal systems that are characteristic of adult workers and are also present in the original developmental pattern. High levels of JH may, for instance, inhibit the development of worker-specific leg structures [59], as well as prevent cell death in the ovaries, leading to a higher number of ovarioles in adult queens. Moreover, JH is likely to control the extended brain development specifically that of MB, resulting in bigger worker brains when compared to queen brains [26,2]. Thus, JH has contrasting effects on growth and development, at least on certain structures and might be regulating trade-off processes during caste differentiation in *A. mellifera*.

Supporting our general model, the genes in the queen putative regulatory network are mainly associated with motifs discovered in the hormone responsive dataset (Figure 3). In contrast, the genes (*usp, crc*, *RfaBp *and *actin*) in the worker network are mainly connected via worker- and apoptosis-biased motifs.

Several steps of the proposed pathway leading to caste development have better experimental support than others. Without doubt, the proximate mechanism(s) linking Tor activity and JH synthesis (a general biological issue) are still unclear. We also have to investigate whether an ALS-like mechanism of systemic communication of the organism's nutritional condition is functional in honeybees (the *A. mellifera *ortholog of *als *gene is GB20133). We further need to deepen our understanding of a number of molecular pathways that are critical for the establishment of caste identities, including the development of the nervous system, ovaries and legs. Finally, silencing key genes like *usp, crc *and *RfaBp *in developing workers and *tor *in queens would be particularly advantageous in gaining novel insights into the behavior of the proposed regulatory networks.

## Methods

### Overview

Two types of spotted microarrays were used in this study. A custom-made small array contained 768 ESTs implicated in processes believed to be important for caste determination; 723 of these ESTs were manually selected from the ORESTES project [19] and 45 were arbitrarily chosen from the existing ESTs encoding transcription factors and microRNAs. The small array was produced in the Adelaide Microarray Facility. The second array representing brains of mixed age workers was constructed by Robinson and colleagues (bEST_9000; University of Illinois at Urbana-Champaign, USA; [60]). Together, these slides account for more than 60% of the honeybee genes.

The differential feeding between the prospective queens and workers begins after larval stage 3, when the nurse bees continue to feed queen larvae with large quantities of royal jelly, whereas they include honey and pollen in the worker larvae's diet, reducing the amount of royal jelly. This switch in feeding is somehow linked to a differential JH synthesis determining higher titres of this hormone in queens than in workers's hemolymph [5]. To identify genes that are responsive to these hormonal and nutritional changes we tested RNAs from third instar (L3), fourth instar (L4) and fifth instar spinning stage larvae (L5S2), characterized by high (L3–L4) and low levels of JH (L5S2).

As JH is known to govern the induction of queen development in highly eusocial bees, we also tested RNAs coming from worker larvae treated with JH-III in L4 (samples obtained 1 h and 24 h after hormone treatment). These hybridizations could give us information about the genes responsive to exogenous JH, whose profiles could be compared to those obtained in normal L3–L4 queens and thus, highlight those genes regulated by JH and responsible for the development of prospective queens.

### Beekeeping and hormone treatment

Honeybee larvae were collected from *A. mellifera *colonies (Africanized hybrids) maintained at the Experimental Apiary of the University of São Paulo at Ribeirão Preto, Brazil. Larvae of the same and determined age were obtained as in Barchuk et al. [61]. The developmental stages were classified according to the criteria proposed by Michelette and Soares [62].

To test the effects of exogenous JH on worker larvae gene expression profile, fourth instar larvae received a topical application of 10 μg JH III (C_16_-juvenile hormone, Fluka Biochemika, 59992; diluted in acetone to a stock solution of 2.5 μg/μL). The amount of applied hormone was based on our previous experiments in which we examined the induction of *vg *and *usp *expression by JH during post-embryonic development in honeybees [61,39] and on a pioneer work of Goewie and Beetsma [63] of "artificial" queens development by JH application. JH-applied larvae (n = 80–100) in brood frames were left in an incubator (34°C and 80% relative humidity) for 1 h previous putting them back into the colony where they were maintained for 24 h. After the appropriate time, larvae (JH treated and control) were included in TRIzol reagent (Invitrogen) and frozen at -80°C until RNA extraction.

### Microarray preparation and hybridization

Microarray experiments are described according to the MIAME specifications [64] and the resulted data have been deposited in the Gene Expression Omnibus (GEO, at NCBI database) under the accession number GSE6452 [65]. The 768_EST microarrays were prepared from PCR amplified cDNA clones using either M13 or specific primers. The amplicons were washed and EtOH precipitated before preparing the final samples for spotting. cDNA fixation on glass supports was done by the Adelaide Microarray Facility [66]. Prior to hybridization each slide was incubated in pre-hybridization solution (10 mg/mL BSA, 25% formamide, 5× SSC and 0.1% SDS) for 60 min at 42°C then rinsed in Milli-Q water and dried by centrifugation at 750 rpm for 5 min.

We used 30 arrays (768_EST and bEST_9000) for both kinds of experiments: developmental gene expression and JH-responsive genes evaluation. For internal controls we used heterologs or reference genes like phosphoglycerate kinase 1 and 2-microglobulin (cattle), rubisco small chain 1 and chlorophyll ab binding protein (soy; see [60]) and ribosomal protein S8 (honeybee). Each slide included two (768_EST) to four (bEST_9000) replicates. Pairs of RNA samples (labeled with Cy3 or Cy5 fluorophores) from prospective queens and workers from each developmental stage (L3, L4 and L5S2) and JH-treated and control workers were hybridized to the same slide. Dye swaps were done for each comparison and more than two slides were used to evaluate some developmental stages.

Total RNA was extracted following Invitrogen's protocol combined with column purification (RNeasy Mini Kit, QIAGEN, Cat. 74104). RNA quality was determined by electrophoresis in agarose gels as in Kucharski and Maleszka [67]. cDNA synthesis, RNA amplification and probe synthesis (from 1 μg of amplified RNA) were done with and according to Low RNA Input Fluorescent Linear Amplification Kit (Agilent, Cat. 5184-3523). Probes purification was done using MinElute PCR Purification Kit (QIAGEN, Cat. 28004).

For hybridization, probes (in 90 μL 2× SSC) were pre-heated at 50°C during 3 min and placed on microarrays under lifter-slip cover glasses (22 × 60, 31.25 μL). cDNA microarrays were placed in single slide hybridization chambers which were incubated in a water bath for hybridization for 17h, at 50°C. Washing procedure included the following steps: 2× SSC and 0.1% SDS; 2× SSC; 0.1× SSC and bidistilled water; 3 min per wash, all at room temperature. Slides were dried by centrifugation at 750 rpm for 5 min previous scanning.

### Raw data obtaining and analyses

Slides were scanned using Affymetrix 428 Array Scanner and Jaguar 2.0 software; gain 40–68 dB, 10 micron resolution, Cy3 with Green Laser (532 nm), Filter FM570-10 and Cy5 with Red Laser (635 nm), Filter FM665-12. For images quantitation we used ScanAlyze 2.35 [68] with default parameters.

Background correction was performed to avoid the correction of negative or zero intensities (offset correction) by adding to the background-corrected intensities a positive constant (= 50). It damps spurious variation in log-ratios, particularly at low intensity spots. The "print-tip loess" normalization was used to correct for within-array dye and spatial effects and single channel normalization was used to facilitate comparison between arrays [69]. Since our slides had 4 (768Br) and 2 (bEST_9000) within array replicate spots, the normalization procedure also considered the variation among their values [70]. After normalization we determined a log_2 _ratio (Queen's sample intensity/Worker's sample intensity), for each probe on each array. The fold-change in expression and its standard error for each gene were calculated by fitting a linear regression to the normalized expression data. A Bayesian smoothing procedure was used to shrink the estimated standard errors, with which we calculated the moderated *t*-statistic for each gene [69].

Differentially expressed genes (ESTs) from 768_EST and bEST_9000 slides were assigned to GB IDs of the GLEAN3-predicted protein sequences [71]. All DEGs were annotated according to Gene Ontology terms for Biological Process and Molecular Function [72]. FatiGO web tool [73] was used to annotate Biological Process and Molecular Function terms based in *D. melanogaster *sequence similarity for the target genes matched by the overrepresented *cis *elements.

### Computational analysis of upstream regions of differentially expressed genes

In order to detect biologically relevant motifs (*cis*-elements) in the upstream control regions (UCR) of the sets of JH- and caste-related genes we selected the input data set based on two different criteria: (1) those genes that have shown significant differential expression between castes and in hormone assays observed in the microarray analyses, and (2) those genes differentially expressed in castes that shared functional similarity with empirical evidence already described in the literature (Tables 1, 2, and Additional file 1). A motif discovery script was designed based on reliable strategies proposed by MacIsaac and Fraenkel [74] and Cristino et al. [14]. The pipeline run separately on the two main sets of UCR sequences combining the output of three programs: AlignAce [50], MEME [51] and MDscan [52]. Default parameters values were used in all searches, except that GC-content background in intergenic regions was set to 25% for AlignAce and a background distribution file was computed for MDscan.

A database containing 10,123 UCR sequences was generated by parsing the Official Gene Set annotation file assembly 3.0 (downloaded in GFF format from 75) to extract upstream regions starting from the terminal 5' genomic coordinate of each predicted CDS. The UCRs were arbitrarily set to a size frame of 1000 nucleotides, but were trimmed whenever another predicted ORF was detected in any of these regions [50].

MAP (maximum *a priori *log likelihood), group specificity score (Church score) [76], ROC-AUC (area under the curve for a receiver operator characteristic plot) metric and MNCP (mean normalized conditional probability) metric [77] were used to score the discovered motifs. Motifs databases were generated for each subset of genes with MAP score equal to and greater than 5.0. A non-parametric test (Kolmogorov-Smirnov two-sample test) was conducted to infer significance levels for the 19 set of discovered motifs. The distribution of the four motif's score metrics for all 19 set of (1) hormone- and caste-differentially expressed genes and in (2) functional groups up-regulated in castes [see Additional file 2] was tested against motifs discovered in control random dataset. Control UCR dataset were 100 times randomly selected from the genomic background (10,132 UCRs) equal in size to each 19 input set and control motifs were generated by running the motif discovery script in randomly selected sequences. Only motifs with very stringent scores (MAP ≥ 5; Church ≤ 1e-10; ROC-AUC ≥ 0.7) were considered in this manuscript.

Functional ecdysone response elements have been identified and it is now well established that the EcR-USP complex binds to direct or inverted (palindromic) repeats (rGkTCAATGaMCy) [78,55,79]. Another binding site pattern involving USP (CF1-USP) was already identified in *D. melanogaster *s15 chorion gene (GGGGTCAcs) [54]. Both known motifs were searched in all 183 DEG.

The discovered and known motifs were represented by position weight matrices (PWM) [80,81]. Each bee motif was aligned against the *D. melanogaster *sequences in the TRANSFAC database (release 4.0) [53]. Only the alignments with a threshold of 80% identity for each PWM were considered as significant matches.

The interrelation between overrepresented motifs and genes was modeled as a bipartite network based on concepts from graph theory [82] and complex networks [83]. The clustering coefficient (cc) and degree (d; 83) were calculated from subsumed networks, which were obtained from the respective bipartite representation by linking all pairs of genes connected to a same motif, thus resulting in networks with only one type of node.

An Ubuntu Linux (version 6.06; 84) operating system was used to implement all scripts and pipelines designed for annotation procedures and motif discovery. The Python programming language [85], Biopython [86], and TAMO (Tools for Analysis of Motifs) packages [87] were used in program design. For the microarray analyses, all normalizations and fold change calculations were performed using the functions in the library *Limma *of the R/Bioconductor package [88]. For the detection of conserved domains, the 84 protein sequences used to motif search were screened against the Pfam database [89] using the HMMER platform (current release 2.3.2; 90), with a cutoff value set at 1e-5.

## Authors' contributions

ARB, ZLPS and RM designed research; ARB and RK performed research; ARB coordinated the collaboration and drafted the manuscript; AdSC performed the bioinformatics analyses and participated in drafting the manuscript. LdFC made the statistical analyses of the networks and supervised AdSC. ZLPS supervised ARB and AdSC and helped drafting the manuscript. RM supervised ARB and RK and participated in writing the manuscript. All authors read and approved the final manuscript.
